# Intestinal obstruction secondary to perforation of Meckel’s diverticulum caused by dentures: a case report and review of literature

**DOI:** 10.1186/s40792-024-01959-x

**Published:** 2024-06-21

**Authors:** Gaoyuan Tian, Zefeng Yuan, Ming Luo, Yujin Zhang, Bin Kong

**Affiliations:** Department of Gastrointestinal Surgery, Hebei Medical University Third Hospital, 139Ziqiang Road, Shijiazhuang, 050051 Hebei People’s Republic of China

**Keywords:** Swallowed denture, Meckel’s diverticulum, Intestinal perforation, Intestinal obstruction, Exploratory laparotomy

## Abstract

**Background:**

Meckel’s diverticulum (MD) is the most common congenital abnormality of the gastrointestinal tract. However, MD is rare in clinical practice, and perforation of a MD by a foreign body is even rarer. Preoperative diagnosis is difficult because there is often insufficient information; therefore it is usually diagnosed intraoperatively. Although rare, it should be considered as a differential diagnosis in patients who have ingested foreign bodies.

**Case presentation:**

The following is the case of a 52-year-old female patient who was admitted because of generalized abdominal pain for 5 days, related to nausea and vomiting. She also stopped passing gas. Inflammatory indicators were elevated, and computed tomography (CT) revealed gas–liquid levels in the small intestine and high-density objects in the ileum. Based on the patient’s condition, laparotomy was performed instead because the laparoscopic procedure was difficult to perform. Intraoperatively, a foreign body perforated the diverticulum of the terminal ileum, resulting in the development of an abdominal abscess. Finally, we performed resection of the ileal diverticula and partial resection of the ileum. After the surgery, it was confirmed that the foreign bodies were two dentures accidentally eaten by the patient.

**Conclusion:**

A thorough understanding of the clinical presentation, imaging features, and treatment of MD and its complications will assist clinicians in making prompt and accurate diagnoses and providing symptomatic treatment.

## Background

Meckel’s diverticulum (MD) is the most common congenital abnormality of the gastrointestinal tract. Perforation of a MD by foreign bodies is a rare complication that lacks clinical specificity and is easily misdiagnosed. Existing imaging methods are rarely helpful for the preoperative diagnosis of diverticula. Clinicians must perform a comprehensive assessment through a detailed history, physical examination, and imaging examination. Surgical resection is generally accepted as the treatment of choice for symptomatic MD. When dealing with MD and its associated complications, surgeons should choose between laparotomy or laparoscopic surgery based on the specific circumstances of the patients.

## Case presentation

**Fig. 1:**
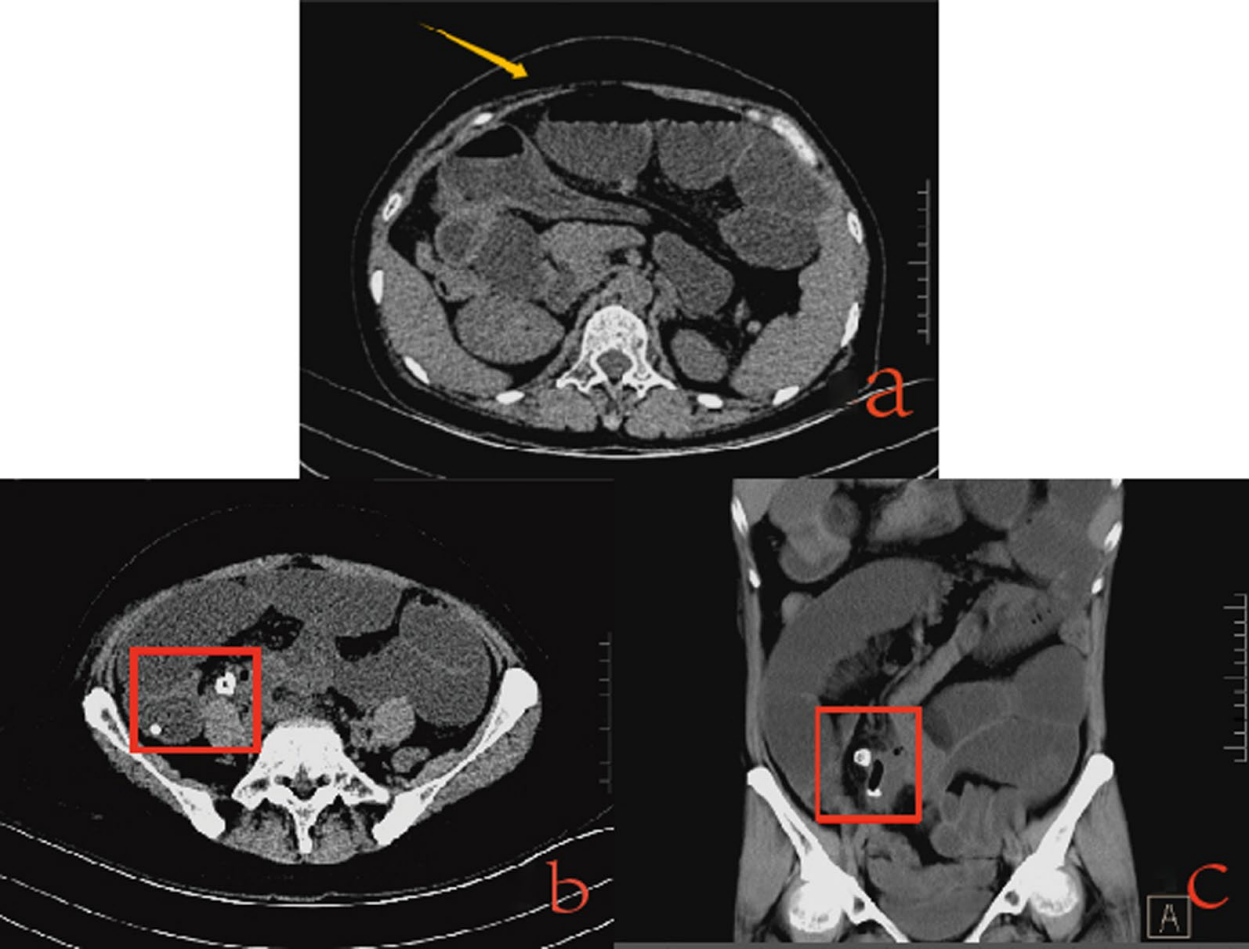
a Computed tomography (CT) of the abdomen showed partial small bowel dilatation with **a** gas–liquid level inside. **b** Cross section Computed tomography (CT): Increased density of the local intestinal canal in the right lower quadrant. **c** Coronal Computed tomography (CT): increased density of the local intestinal canal in the right lower quadrant

A 52-year-old woman presented with persistent generalized abdominal pain 5 days prior to presentation. The patient vomited after eating. The vomit consisted of stomach content. She stopped passing gas and had only a small amount of loose stools. The patient had no chills or fever. On clinical evaluation, the vital signs were stable. Flat abdomen, no gastrointestinal pattern or peristaltic waves. During palpation, abdominal tension, mild tenderness, and rebound pain were observed in the right lower abdomen. Hypoactive bowel sounds were detected during the auscultation. Initial laboratory workup revealed a normal white blood cell count but an increased C-reactive protein level of 236.04 mg/L. The percentage of neutrophils (NEUT%) increased to 85.5%. These findings suggest an inflammatory response in these patients. Computed tomography (CT) of the abdomen showed partial small bowel dilatation with a gas–liquid level inside (Fig. 1a). Increased density of the local intestinal canal in the right lower quadrant, increased density of the fat space around the intestinal canal, and unclear display of fine structures were observed (Fig. 1b, c). After completing the imaging examination, we continued to inquire about her relevant medical history. She admitted that she had a history of losing dentures before developing abdominal pain. Based on the patient’s clinical manifestations and auxiliary examinations, we speculated that the presence of foreign objects in the patient’s intestine may be her dentures, which may cause intestinal obstruction or even perforation, leading to local inflammatory reactions. We cannot rule out the possibility of a diverticula in the distal ileum. Before surgery, the patient was given gastrointestinal decompression, but the effect was not significant, and abdominal pain still persisted. Finally, we chose surgical treatment.

Preoperative diagnosis: 1. intestinal obstruction 2. Foreign body in small intestine

**Fig. 2:**
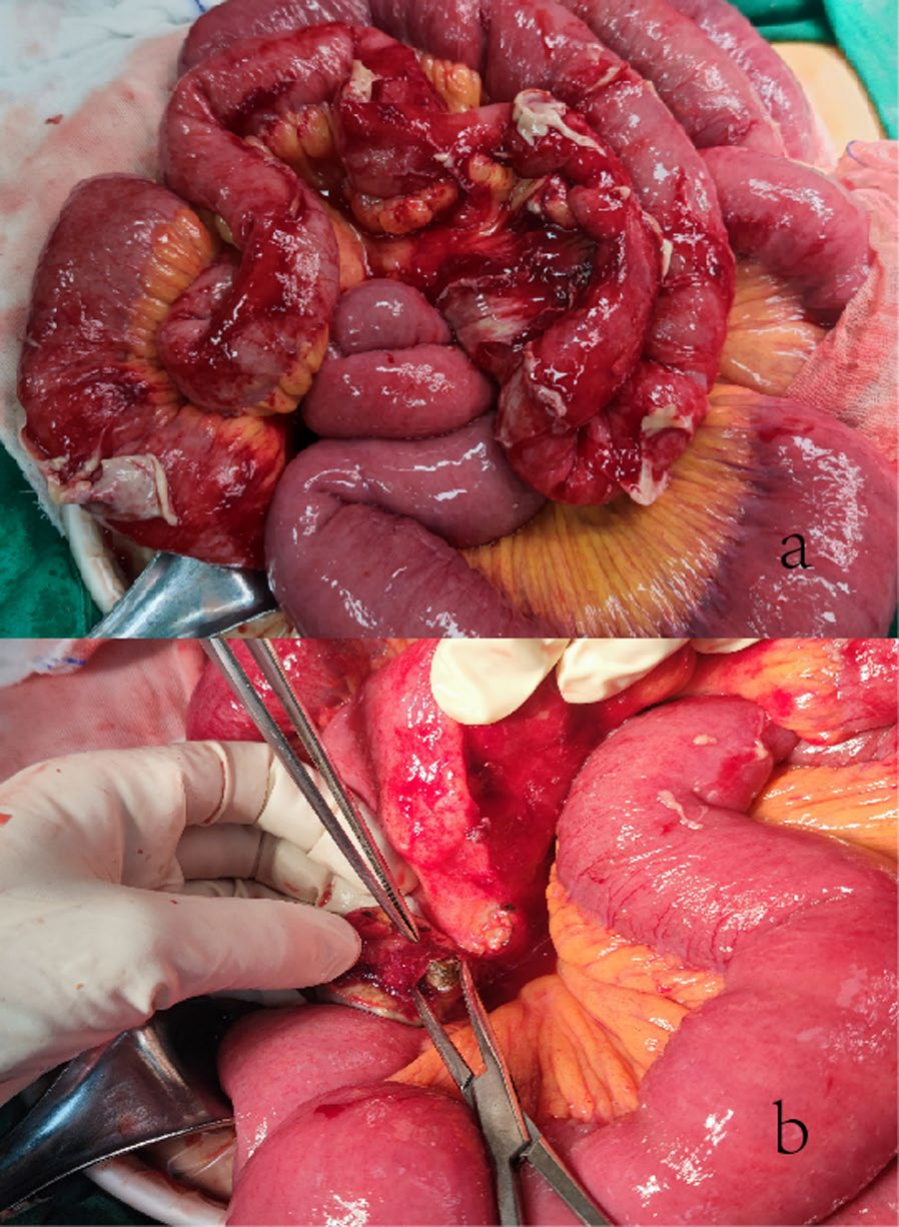
**a** Exploratory laparotomy revealed significant dilatation of the proximal small intestine and adhesions of the distal small bowel to the pelvis. An abscess developed in the right lower abdomen of the ileocecal region. **b** A hard metallic foreign body was removed from the diverticula

After obtaining informed consent from the patient, she underwent a laparoscopic exploratory surgery. Laparoscopic exploration revealed significant dilation of the small intestine and severe pelvic adhesions, which made laparoscopic surgery difficult. As a result, we decided to enlarge the abdominal incision and perform a laparotomy. Exploratory laparotomy revealed significant dilatation of the proximal small intestine and adhesions of the distal small bowel to the pelvis. An abscess developed in the right lower abdomen of the ileocecal region (Fig. 2a). We divided the adhesion and drained the abscess. There was a hard metallic foreign body in the abscess cavity. Diverticula can be observed in the distal ileum near the ileocecal valve. The cavity wall consists of a part of the diverticula wall with local ischemia and perforation. A hard metallic foreign body was removed from the interior (Fig. 2b). We separated the mesangial tissue surrounding the diverticulum and excised it. We then separated the adhered intestine and found angulated adhesions of the ileum approximately 60 cm from the ileocecal valve and poor blood circulation in the bowel of approximately 20 cm. We then performed resection of the diseased bowel and ileoileal anastomosis. Finally, negative pressure drains were then placed and secured in the subhepatic, splenic fossa, pelvis, and subcutaneously. The surgery lasted for 3 h, with intraoperative bleeding of 100 ml.

**Fig. 3:**
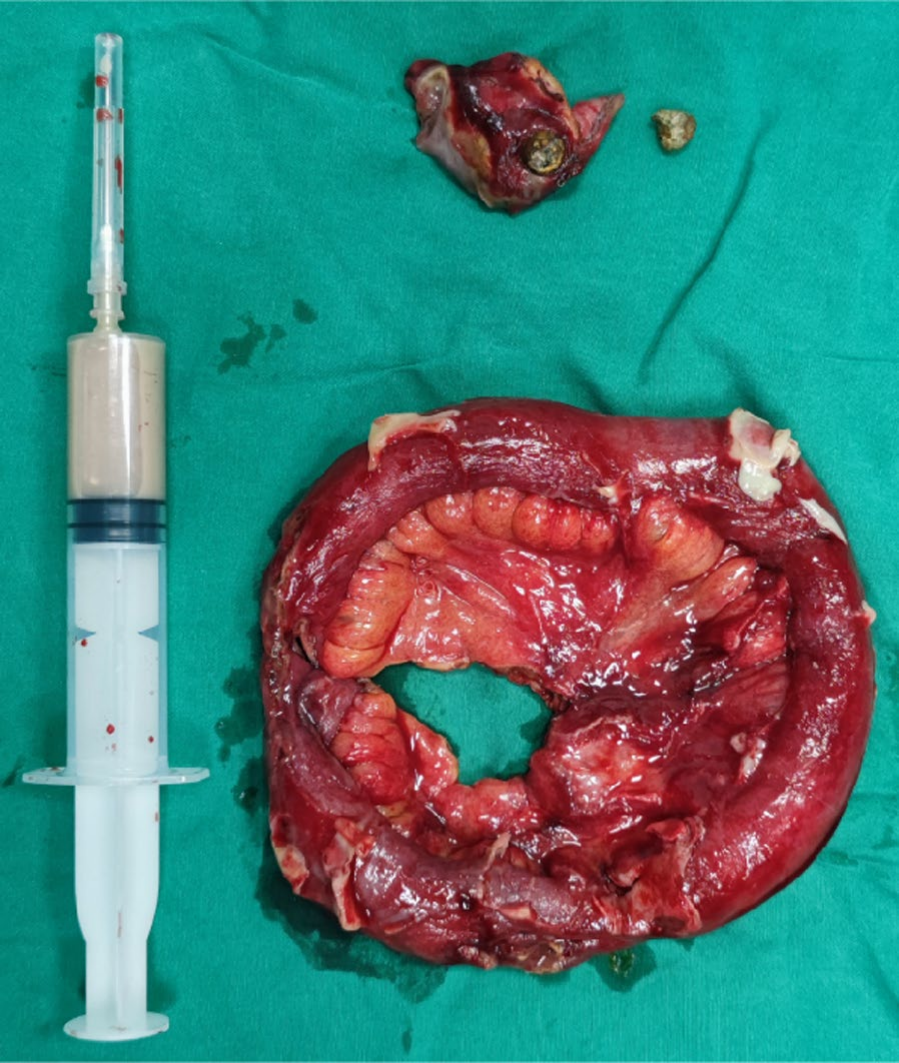
Surgical specimen: A segment of the ileal intestinal canal, about 20 cm in length; a diverticulum in ileum, about 3.5 cm * 3 cm * 1 cm in size; pus was drawn from the abdominal abscess, about 8 ml; two metallic foreign bodies

**Fig. 4:**
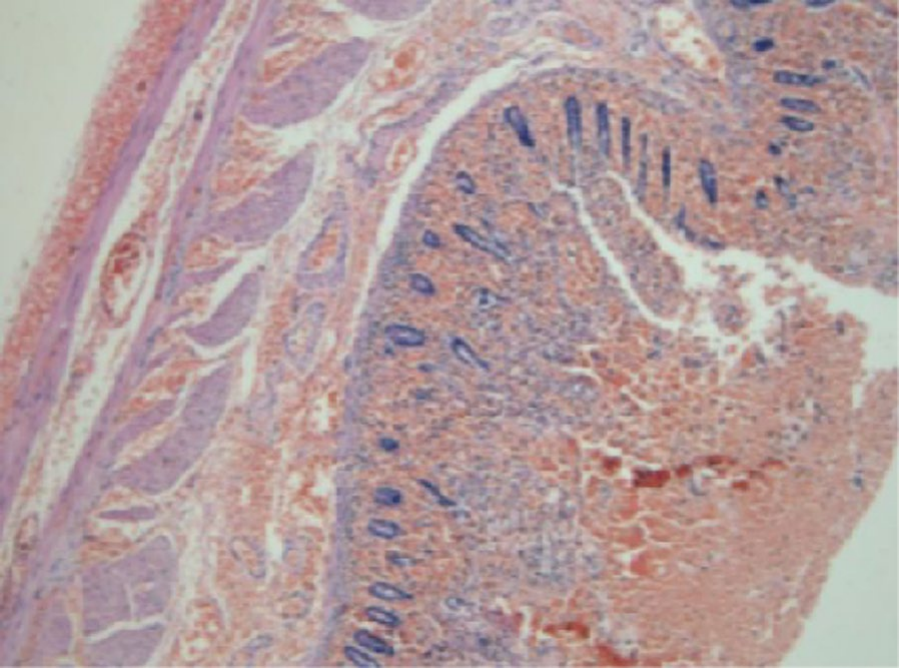
Pathology confirmed that we were facing a Michael’s diverticulum with all layers of the intestinal wall

Surgical Specimen: A segment of the ileal intestinal canal, about 20 cm in length; a diverticulum in ileum, about 3.5 cm * 3 cm * 1 cm in size; pus was drawn from the abdominal abscess, about 8 ml; two metallic foreign bodies (Fig. 3). Pathology confirmed that we were facing a Michael’s diverticulum with all layers of the intestinal wall (Fig. 4). The patient safely returned to the ward after surgery, passed flatus and defecation on postoperative day 3, started a liquid diet on postoperative day 7, and was discharged on postoperative day 10. Follow-up was conducted one month after surgery, and the patient reported good recovery without any discomfort.

## Discussion

### Concept of Michael’s diverticulum

The Meckel’s diverticulum (MD) is a finger-like protrusion on the wall of the distal ileum. It is a congenital malformation that results from incomplete vitelline duct degeneration. MD is the most common congenital abnormality of the gastrointestinal tract [1]. Its incidence is 2 to 4%. MD is a true diverticulum that contains all the layers of the intestinal wall [2]. The location of the MD is uncertain, but it is usually found within 100 cm of the ileocecal valve [2]. The classic diagnostic criteria for MD are as follows: the diverticulum has to be located on the antimesenteric border, within 2 ft proximal to the ileocecal valve, contains all five layers of the small intestine, and has its own blood supply [3]. In the present case, pathology confirmed that we were facing a true diverticulum with all layers of the intestinal wall. The 3.5 cm long and 3 cm wide diverticulum was located on the distal ileum 30 cm from the ileocecal valve.

### Meckel’s diverticulum and its complications

MD is asymptomatic in most affected individuals, with a 4.2–16.9% probability of symptomatic presentation [4]. The clinical presentation ranges from intestinal obstruction to bleeding, inflammation, and perforation [5]. While children with MD more often present with gastrointestinal bleeding, intestinal obstruction is the most common presentation in adults. Perforation of the MD is extremely rare [5]. Most often when fecaliths obstruct the diverticulum, leading to inflammation and necrosis. More rarely, perforation is due to foreign body perforation. Foreign bodies, including fish bones, gallstones, enteroliths, marbles, bullets, and phytobezoars, have been reported in less than 2% of symptomatic MD. The foreign bodies that cause perforation of MD are more often sharp objects, such as fishbones and date pits [6]. In this case, the patient accidentally ingested dentures, which perforated MD, resulting in an abdominal abscess. This unexpected complication eventually led to intestinal obstruction. Edentulous individuals are also at a higher risk of ingesting foreign bodies, including dentures, owing to reduced sensation in the oral mucosa and poor motor control of the laryngopharynx [7]. Another important issue that increases the risk of denture ingestion is the lack of patient awareness regarding the need for regular check-ups and denture changes or compliance [8]. It is worth mentioning that fixed dentures, as well as removable dentures, can be accidentally ingested. Therefore, patients wearing dentures should be advised to regularly review their condition, and patients with loose dentures should see a dentist as soon as possible.

### Imaging diagnosis of Meckel’s diverticulum

Imaging has limited value in the diagnosis of MD. Only 10% of symptomatic MD are definitively diagnosed preoperatively [5]. Acute appendicitis is the most common cause of misdiagnoses. Plain X-ray, barium studies, and computed tomography (CT) scans are seldom beneficial for the preoperative diagnosis of diverticulum. They can be normal or show nonspecific changes [5]. It is often difficult to make an accurate judgment before surgery, and clinicians must assess it in conjunction with the patient's medical history and clinical symptoms. As in the present case, the diagnosis was only made intraoperatively. Although the diagnosis of MD has posed challenges for surgeons, new diagnostic methods, such as capsule endoscopy, double-balloon enteroscopy, and 99mTc pertechnetate scintigraphy, have emerged in recent years [9, 10]. With further verification of the feasibility of these tests, the diagnosis of MD has greatly developed.

### Treatment of Meckel’s diverticulum

It is generally accepted that resection is the treatment for symptomatic MD. Although exploratory laparotomy has traditionally been the standard approach, it has been shown that laparoscopic approaches have equivalent outcomes and may reduce the overall length of stay [11]. In a study based on the analysis of the NSQIP Pediatric database comparing laparoscopic, laparoscopic converted to open, and open resection in 681 pediatric cases, Skertich et al. demonstrated low rates of postoperative complications and few significant differences between laparoscopic and open surgery. The most common complications observed were surgical site infections, bleeding, and readmission. In addition, the conversion rate from laparoscopic to open surgery was high (27%) [12]. Therefore, when dealing with acute abdomen caused by foreign bodies that require surgical treatment, surgeons should choose between laparotomy or laparoscopic surgery, according to the specific circumstances. In this case, the swelling of the small intestine and adhesion of the pelvic cavity led to a final change in the surgical plan.

When selecting between diverticulectomy or segmental bowel resection with re-anastomosis for resection of MD, it should be based on the morphology of the diverticulum and the condition of the surrounding ileum. Generally, simple diverticulectomy is recommended as long as the base of the diverticulum is small relative to the ileum and there is no inflammation or perforation at the base [13]. Segmental resection of the ileum containing MD is recommended if the base is broad or if diverticulectomy results in significant luminal narrowing. Ileal resection is also recommended if the base is inflamed, perforated, or has a bleeding ulcer [14]. Brungardt et al. analyzed 506 adult cases in the NSQIP database comparing diverticulectomy with segmental resection and found similar rates of complications and mortality within 30 days. Additionally, the most common complications in both groups were readmission, sepsis, wound infection, and reoperation [15]. In the present case, we decided to perform diverticulectomy and partial ileectomy because ischemia and perforation of the local diverticulum wall occurred, part of the ileal adhesions were angulated, and blood circulation in the bowel was poor.

## Conclusion

We experienced a rare case of intestinal obstruction secondary to Meckel's diverticulum (MD) perforation caused by dentures. Due to the difficulty of laparoscopic exploration, the abdominal incision was expanded and converted to open surgery. Our timely surgery helped to control the infection in the abdominal cavity without further deterioration. MD perforation caused by foreign body ingestion is a very rare phenomenon, and its clinical presentation lacks specificity and is easily misdiagnosed. Clinicians need to make a comprehensive judgment through detailed history, physical examination, and imaging examination, in order to receive timely surgical intervention for treatment.

## Data Availability

The dataset supporting the conclusions of this article is available in the manuscript.

## Abbreviations

MD: Meckel’s diverticulum
CT: Computed tomography
